# Coping among Afghan Former Unaccompanied Refugee Children in the UK: A Qualitative Study Exploring Barriers and Influences Over Time

**DOI:** 10.1177/13634615261435699

**Published:** 2026-05-13

**Authors:** Rebecca Lane, Hannah Taylor, Sheila Melzak, Imogen Rushworth, Mazda Beigi, Kenny Chiu

**Affiliations:** 1 Department of Clinical Psychology and Psychological Therapies, 6106 University of East Anglia, Norwich, UK; 2 8953 Norfolk and Suffolk NHS Foundation Trust, Norwich, UK; 3 593883 The Baobab Centre for Young Survivors in Exile, 6 Manor Gardens, London, UK; 4 Beigi & Chiu Clinical Psychology, 71–75 Uxbridge Road, Ealing, London, UK

**Keywords:** unaccompanied refugee minors, coping, resilience, psychological well-being, Afghanistan

## Abstract

Unaccompanied Refugee Minors (URMs) are an extremely vulnerable refugee group, at heightened risk of adversity and trauma, as well as long-term, severe mental health difficulties. There is a lack of research providing a contextual understanding of difficulties and active resilience processes to inform how to promote URM wellbeing. This qualitative study explored the difficulties and coping responses of former URMs from Afghanistan and perceptions of how and from where coping strategies are developed. Reflexive Thematic Analysis of 12 interviews generated three overarching themes: “*Difficulties and coping: a series of cumulative challenges and vicious cycles*”; “*Roots to coping*”; and “*Coping is dynamic: the process of change*”. Participants reported multiple and compounding difficulties, often present in a triad of physical/psychosomatic pain, mental health difficulties and social challenges. Participants described resilience mechanisms and the barriers to coping strategies being used and effective, revealing how the ability to employ strategies may be limited and that strategies may contribute to further challenges. Experiences of early attachment, culture and identity could be observed to influence the ways coping strategies are appraised and developed. The importance of prioritising belonging and providing trauma- and attachment-informed care is discussed.

Numbers of children who seek asylum unaccompanied and separated from parents or guardians, or Unaccompanied Refugee Minors (URMs), have increased in the UK (Department for Education, 2023). URMs are among the most vulnerable refugee populations, experiencing loss of key attachment figures and abstract losses of safety, culture and context. Due to family separation and young age, URMs are at heightened risks of harm before, during, and after migration (Fazel et al., 2015; Von Werthern et al., 2019). In comparison to accompanied refugee children, URMs are more likely to experience multiple traumas (Fazel et al., 2015), and suffer from severe mental health difficulties, including Post-Traumatic Stress Disorder (PTSD) and depression (Bean et al., 2007; Höhne et al., 2023). In the UK, URMs are supported by a network of services spanning local authority, education, National Health Service and third sector organisations. However, significant barriers to accessing appropriate support among refugees and asylum seekers are known to exist (Asgary & Segar, 2011; Byrow et al., 2020; Lebano et al., 2020; Pollard & Howard, 2021; Satinsky et al., 2019), resulting, for example, from age disputes, language differences, discrimination or mental health stigma. Research providing a contextual understanding of such difficulties and protective factors is urgently needed to inform clinical practice and enhance URM wellbeing (Sleijpen et al., 2016).

Not enough is known of URM promoters of resilience (Mitra & Hodes, 2019), conceptualised as “*cumulative intra-individual traits and/or contextual factors that promote physical or mental well-being in the presence of adversity*” (Rodriguez & Dobler, 2021). A recent meta-analysis found a significant link between resilience and anxiety, depression, PTSD, and psychological distress among forcibly displaced populations (Lane et al., 2025). This review notes a lack of resilience and mental health research involving URMs. It also underscores the challenges of defining resilience in research, as it is multi-faceted and subject to various interpretations, consistent with broader literature on resilience (Davydov et al., 2010) and mirroring challenges experienced by refugee populations in defining “resilience” (Hawkes et al., 2021). Under the broader umbrella of resilience, sits coping, representing an active and conscious process by which resilience buffers distress. Studies exploring this tangible aspect of resilience (Rice & Liu, 2016) may shed light on how individual and environmental impacts of stress can be mitigated (Frydenberg, 2014), and consider how coping can be promoted.

Research exploring how URMs cope has noted strategies of avoidance and distraction, adopting positive outlooks, social connection, and religiosity (Behrendt et al., 2023; Ní Raghallaigh & Gilligan, 2010). However, these studies often recruit culturally heterogenous samples and rarely explore resilience processes in culturally specific groups (Sleijpen et al., 2016), limiting present understanding of the influence of culture on coping, despite the belief that responses to stress and coping strategies may be shaped by cultural and religious norms (Ungar, 2006, 2008). To the best of the authors’ knowledge, only two UK studies include URMs from one cultural background (Chase, 2021; Nasir, 2012). They focus on adaptation to living in the UK, navigating asylum processes and the experience of entering adulthood, rather than considering difficulties and coping more broadly. In addition, no research to date has sought to understand how individual coping strategies are developed among URMs, as well as what might impede coping strategies being employed.

Research on resilience and coping underscores the critical role of early attachment relationships (Darling Rasmussen et al., 2019). Among young refugees, quality of parenting and early relationships have been associated with mental health outcomes (Scharpf et al., 2021; Scharpf et al., 2023), with attachment patterns potentially mitigating the effects of trauma (Bettmann & Olson-Morrison, 2021; Eruyar et al., 2020). Maternal attachment has been found to moderate the effects of resilience building interventions for accompanied refugee children (Diab et al., 2015) and predict positive outcomes of psychological interventions (Punamäki et al., 2021). Parental separation and rejection at times of forced migration have been highlighted to affect levels of resilience among conflict-affected migrants long-term (Siriwardhana et al., 2014), positioning URMs as particularly vulnerable to experiencing challenged resiliency resources and coping strategies. Exploring how coping strategies are developed among URMs would offer new insight for services to draw from. Research has highlighted an overrepresentation of psychiatric and medical lenses through which URM needs are viewed and contextualised (Wernesjö, 2012), noting a need for research gathering URM perspectives. Qualitative research, which allows for in-depth and contextually situated exploration of individual views and behaviours, is called for to better understand how ways of coping are used and perceived to be developed (Sleijpen et al., 2016; Ungar, 2004, 2008).

As the largest group of URMs applying for asylum in the UK in the year ending September 2023 (Refugee Council, 2023) and representing approximately half of URMs applying for asylum in Europe in 2021 and 2022 (European Asylum Support Office, 2022), this study will focus on the perspectives of URMs from Afghanistan. Afghan refugees represent the world's largest protracted refugee population due to decades of war and political instability (Alemi et al., 2023). Documented common experiences of growing up in Afghanistan include exposure to violence and consequences of violence, lack of freedoms and poverty, alongside the mistrust and divisions in communities generated by a range of community conflicts, various militia groups and interventions from external armies (Nascimento et al., 2022; Qamar et al., 2022). Research shows that common family experiences for children in Afghanistan include belonging to families where fear and violence permeate, or having lost or been separated from parents and consequently received no consistent parental care (Catani et al., 2009; Nascimento et al., 2022). Coping strategies among Afghan refugees have been found to be largely embedded in one's faith and family (Alemi et al., 2023). It is important to investigate coping among URMs, for whom cultural dislocation and family separation may prevent them drawing on these areas of coping.

The present study aimed to add to the limited evidence base and explore the difficulties and coping responses of URMs from Afghanistan using qualitative research methods, and to shed light on how and from where coping strategies are understood to be developed. It recruited former URMs (now adults) to enhance capacity for reflection and to capture coping in the context of long-term mental health difficulties (Jensen et al., 2019; Vervliet et al., 2014).

## Methods

### Study Design

This qualitative study employed semi-structured interviews to explore the difficulties experienced by Afghan former URMs, their coping mechanisms and their perceptions of how these are developed. Grounded in a social constructionist perspective (Crotty, 1998), Reflexive Thematic Analysis (RTA; Braun & Clarke, 2022) was used to support the identification and interpretation of patterns within the interview data (Braun & Clarke, 2021a). This approach facilitates inductive, data-driven coding, whilst recognising the central role and the positionality of the researcher in the analytic process (Braun et al., 2019). This study was granted ethics approval by the UEA Faculty of Medicine and Health Sciences Research Ethics Committee (ETH2324-0221).

### Data Collection

Participants were recruited from a service working clinically with URMs or former URMs (now adults) in England, offering psychotherapy, case work and social work, support through the asylum systems, and creative and community activities. All service users have experienced a sequence of violent experiences and human rights abuses, as well as family separation.

Service users were eligible for inclusion if they were aged 18 and above; from Afghanistan; entered or migrated to the UK as a URM; and could communicate in English or through an interpreter. Participants were not eligible for inclusion if they were not deemed to be Gillick competent, were actively suicidal or reported acute psychotic symptoms.

Eligible participants were identified and contacted by a local collaborator using a purposive sampling strategy. If interested, an interview was arranged with the lead author. Participants had not met with the researcher prior to the study but were aware the research formed part of the thesis requirements for her doctoral dissertation. The study aimed to recruit 10–14 former URMs. It is important to note that principles of data saturation, rooted in positivism (Varpio et al., 2017), are not consistent with the study's social constructionist and RTA approach (Braun & Clarke, 2021b).

Once consented into the study, participants completed a demographic questionnaire and took part in a semi-structured interview, conducted online or face-to-face, depending on participant preference. Interpreters were present unless participants requested otherwise. The interview explored areas of difficulties and wellbeing, ways of coping over time, and how coping strategies are perceived to be developed. Example questions include: “*How do you manage when these things happen or come up for you?*”; “*How and from where do you think you learned to cope in this way?*”. The interviews took place between March and September 2023, lasting on average 39 minutes (range: 24–62 minutes). Interviews were audio-recorded and transcribed clean verbatim. Participants received a £20 voucher as compensation and were reminded of available mental health support services.

### Participants

Twelve former URMs from Afghanistan (*M*_age_ = 23.25 years, range: 20–27) were recruited, all identifying as male. Ethnicities disclosed include Afghan, Pashtun, Hazara and Tajik. Participants entered the UK between the ages of 14 and 21 years (mean = 16.17 years) and resided in the UK between 2 and 11 years (mean = 7.08 years). Two eligible participants were approached but declined participation, while scheduling an interview time proved challenging for four others who initially agreed to participate.

### Analysis

The six phases of RTA outlined by Braun and Clarke (2006, 2022) were employed to synthesise and interpret interview data, working across and between the phases in an iterative manner until the findings were written up. Coding and analyses were completed by the first author.

During data familiarisation, the researcher became deeply immersed in the data, transcribing interviews, listening to interview recordings, and re-reading field notes and transcripts. Coding of transcripts, supported by NVivo (QSR International, 2022), deconstructed narratives into discrete semantic and latent units. Coding was inductive, allowing for the findings to be data-led, whilst acknowledging the influence of prior knowledge and researcher subjectivity (Braun & Clarke, 2019, 2021a). Candidate themes were generated from the codes and subsequently developed and reviewed*.* Developing and reviewing themes involved multiple stages of assessing and adjusting the thematic frame, returning to the codes, the transcripts and the research questions. Finally, themes were refined, defined and named, ensuring themes were distinctive and coherent, and written up.

### Reflexivity and Positionality

Social constructionism and RTA both highlight the role of the researcher in the process of generating knowledge, with experiences, characteristics and prior knowledge that will influence interpretations (Braun & Clarke, 2021a; Losantos et al., 2016). It is important to reflect that the first author, who conducted the interviews and completed the analysis, is White European, female, who was undertaking doctoral level study to qualify as a Clinical Psychologist. In addition, the first author holds no personal experience of forced migration. Theoretical frameworks, including attachment (Bowlby, 1969), social learning (Bandura & Walters, 1977) and psychodynamic theories (e.g., object relations theory; Fairbairn, 1963; Winnicott, 1960), offered important lenses influencing interpretations. Reflexivity and introspection of the first author was fostered by using a reflective journal and through discussions in supervision and consultation meetings.

### Efforts to Enhance Rigour and Credibility

This project benefitted from Patient and Public Involvement (PPI), consulting on the design, materials and findings. Feedback from a PPI process involving two former URM service users, alongside project advisory meetings, was used to inform the development of participant facing documents and the interview protocol. The interview schedule was also piloted and well-received.

Participants were offered the opportunity to review transcripts and the analysis. Three clinicians at the recruitment site and two interpreters from Afghanistan who supported with data collection were recruited to a PPI group. Each group member individually met with the first author to provide feedback on the findings and consider how culture may influence communication and interpretation of findings. Although researcher subjectivity is a valued component of RTA (Braun & Clarke, 2023), the PPI consultation process promoted reflexivity and a form of investigator triangulation (Thurmond, 2001), and added to the breadth and depth of the analysis.

Consultation through PPI or advisory meetings allowed for discussions about potential bias and helped the researcher acknowledge important lenses of prior experience, beliefs and theory. The Consolidated Criteria for Reporting Qualitative Research (COREQ) checklist was used for accurate and transparent reporting (Tong et al., 2007).

## Findings

**Figure 1.**
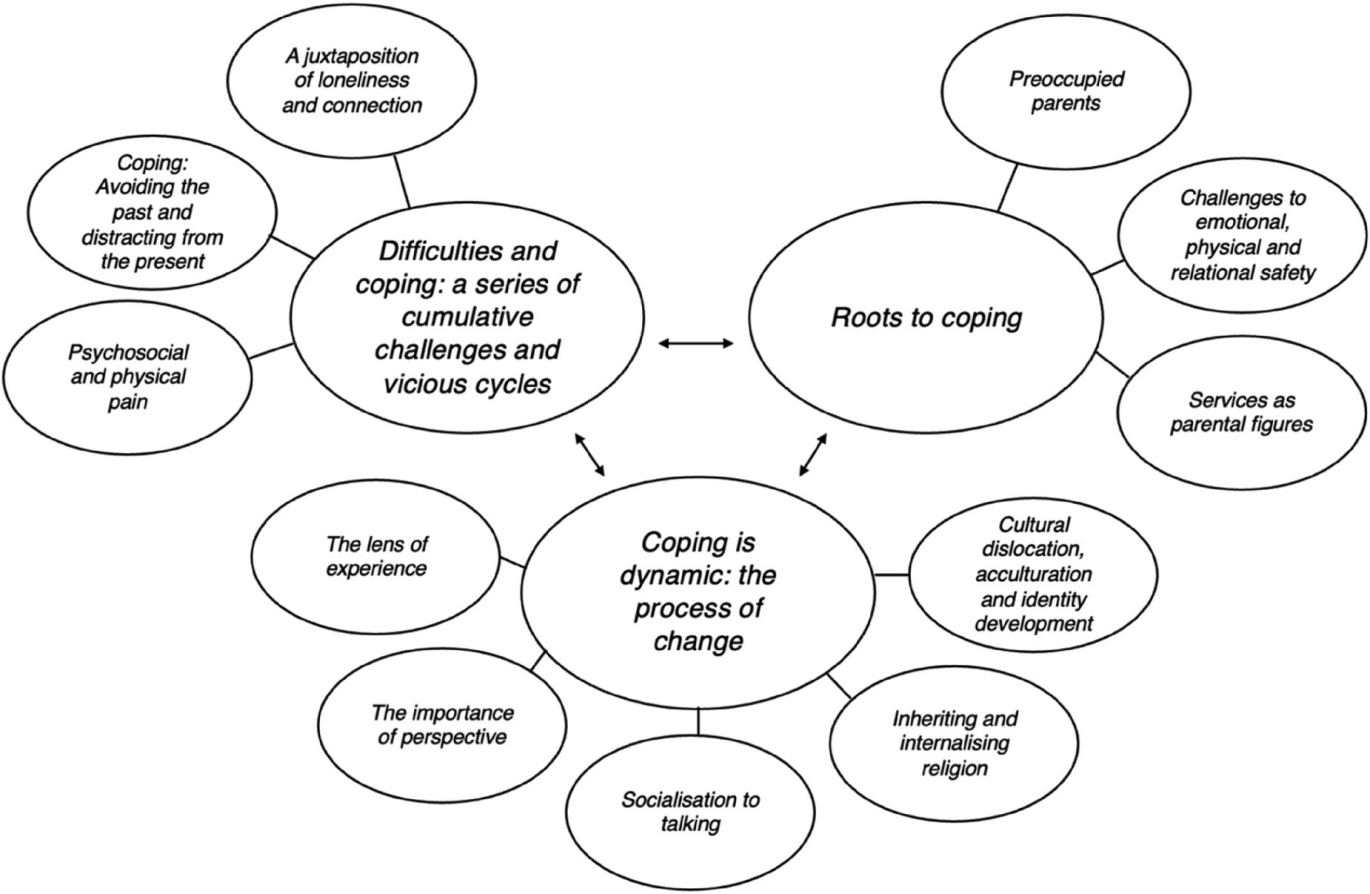
Themes and subthemes.

Three overarching themes were generated from the data: “*Difficulties and coping: a series of cumulative challenges and vicious cycles*”; “*Roots to coping*”; and “*Coping is dynamic: the process of change*” (see Figure 1).

Each theme and associated subthemes are presented below, illustrated by individual accounts in verbatim extracts. Pseudonyms have been used to protect participant anonymity.

### Theme One: Difficulties and Coping: A Series of Cumulative Challenges and Vicious Cycles

This theme describes the types of difficulties participants are faced with day-to-day and how they cope in response, noting a range of challenges experienced that come with or prevent them from employing coping strategies, thus perpetuating difficulties. Considering factors that improve well-being and support with coping was a new exercise for some: “*I haven't talked about anything like that; I haven'’t noticed something like that*” (Ali).

#### Psychosocial, Psychosomatic and Physical Pain

Participants described experiencing a variety of difficulties, most of which revolved around their psychosocial and physical wellbeing: “*I have stress, like I have anxiety, PTSD as well. When I’m alone, I just keep thinking about my family*” (Zahir). These included experiencing intrusive thoughts, worries and memories; overcoming loss; living with shame or guilt; low mood and hopelessness; loneliness; hearing voices; difficulties with memory and learning; difficulties with sleep; and experiences of poor physical health and recurrent pain:

My uncle died, somebody killed him.. […] So that gives me more stress and makes me, like evening sometime when I go sleep, it's in my brain. […] back then I couldn't sleep for one month at all. […] since I came here, I lost a lot of people… like my cousins, they died there. So these things gives you a lot of stress, I am not able to see them. (Habibullah)

These could be amplified by stress due to prolonged immigration processes, education/work or their living environment. For example, Said shared, “*The two things I’m really bothered by are the voices and the pain in my legs.* […] *I also believe* [hearing voices is] *due with accommodation where I live.*” Having to look after themselves was, in itself, a source of difficulty for some, as Nasir shared: “*my thinking from for myself at the moment, it give me stress*”.

#### Coping: Avoiding the Past and Distracting from the Present

Coping with difficulties was largely reported to involve avoidance and distraction. Although being busy could help with achieving goals, for example, education, escaping from the past and difficult feelings were the key drivers. As Asadullah noted, “*if I keep myself busy, then I don’t care much about this loneliness or anything. But if I don’t have a lot to do, then just, you know, these thoughts will keep coming at me*”.

Since I am here, in this country, you know, it's hard. Like whenever I feel low or not good, I’ll always always do something to distract me. Visiting friends, coming to [the therapeutic recruitment service], going swimming, doing exercise, go to [service] or something there. So all these activities, all these things are to keep me busy, so I don’t think about my past. (Jamal)

Physical activity, in particular going to the gym or playing cricket, was reported to be helpful to relieve stress and boost self-esteem. Zahir explained: “*when I was doing gym, so I just completely forget about everything, stop thinking about future. So it's like kind of building yourself, building confidence, you know?*”.

Participants also coped by avoiding problems or people and suppressing their emotions. They reported using drugs, alcohol, and sleep to facilitate avoidance of their stressors. Self-harm was described by a minority as helpful with coping with difficult emotions, distracting from psychological pain:

Sometimes I feel very hopeless and sometimes I feel that life is very meaningless. I feel that sometimes it's best to get drunk and forget about everything. […] I don’t want to go into details but sometimes hurting oneself helps. I felt better and I felt at peace afterwards. (Omar)

Participants spoke of cumulative challenges and double-edged swords which impacted on their ability to employ coping strategies and limited their effectiveness. Some difficulties had an impact on others by decreasing the ability to use strategies for coping, for example, a few participants described a vicious spiral of psychological and physical pain. Said expressed, “*It's a constant struggle. Sometimes, because of the pain in my legs, I find it hard to go out and go somewhere, especially my friends. And if I don’t, I feel even worse*”. Asadullah also queried whether his experience of pain is psychosomatic: “*Sometimes I think maybe some of my pains, it could be all because of the stress that I have, you know? And then that is, you know, translating into physical pain*”.

#### A Juxtaposition of Loneliness and Connection

Social connection was a crux for many participants, who reported being more preoccupied and affected by their difficulties, particularly difficult memories, when they were alone. For example, Yousuf shared “*when I’m home alone, then I know a lot of negative thoughts comes*” and Jamal explained “*the main thing that you know that stresses me out or upsets me is thinking about the past, and being on my own, being lonely. And that loneliness makes me think about the past*”.

Connection was often spoken about in the form of playing cricket, which in addition to enabling social contact, also offered a way to connect to life in Afghanistan:

If I’m at home doing nothing, then I just call my friends, we go out to the park and play some sports, like cricket. […] Since I was 3–4 years old, Yeah, we’ve been playing cricket! […] Back home in Afghanistan – it's like there's no other sports, only cricket. (Farhad)

For some, religious coping was central to their wellbeing: “*I can’t do anything without my religion and without the prayers*” (Farhad) and prayer was described as grounding: “*praying, you know, like just connecting to my creator, it helps me a lot as well. So if I have a lot of stress it just calms me down* […] *It's disconnecting with this world like for 5–10 minutes*” (Asadullah).

A few spoke of the value of having a few good friends, describing relationships in which they feel cared for and where trust and safety is established. Zahir explained, “*best friends, not just friends.* […] *Like family, yeah. So you can share everything with them, they know about everything. They can keep your secret. You know they can help you, you can help them*”*.*

Family's very important to have and having good friends and a network of people that you can actually rely on. You know, reach to them whenever you need to or just even have a nice conversations because I feel, you know, we are social beings and we need to talk to each other. Otherwise isolation will drive us to insanity. (Asadullah)

Talking and sharing feelings were also identified by some as an important way of managing and processing difficult emotions, mostly reported in the context of therapy. As Hamid noted, “*when I talk about what's in my mind and how I feel, it really helps me*”.

However, social contact, although a lifeline at times, was also avoided for fear of worrying loved ones or to avoid participants themselves feeling worried and lonely:

One thing that worries me the most is that one of my daughters and my wife are in Afghanistan and I’m thinking of them, what is going to happen to them and how they are. That upsets me. […] When I am very upset I don’t call them because my mood will affect them as well and upset them. […] Talking to them makes you happy for a couple of days but sometimes it also makes you upset because you see your circumstances and see their problems, that makes you more upset. (Omar)

Some also recognised their need to talk, but noted they did not have anyone to talk to outside of therapy. Hamid described how not speaking English impacts his ability to connect with others and access support: “*because of the language barrier, I can’t even contact* [my therapist] *directly. It's through* [my interpreter] *or my brother I speak to* [my therapist]”.

Although social contact was described as central to wellbeing, many reported ambivalence and barriers born from psychological distress, lack of trust, not feeling understood, and not having the skills or practice to develop friendships as challenges to building or maintaining relationships:

Usually I don’t have any friend, or anything kind of like this. But yes, I was thinking, yes, I want to talk with anyone closely, maybe… Because my contact with people is very bad, I don’t know, I can’t start any relationship or anything with people. It's very difficult for me, I don’t know. About this, I’m thinking it give me very stress. […] But at the moment, not have it or not should fix because my mental [health] is so very bad. (Nasir)

In this vein, some participants contradicted themselves while sharing their experiences, oscillating between truths, which appeared to influence their emotion regulation, appraisals of difficulties, readiness for change and ability to employ coping strategies. For example, as illustrated below, Habibullah spoke about being completely alone and lack of trust, whilst also reporting significant support and love from people close to him:

Nothing makes me happy. […] I don’t trust anyone. There's nobody here. This country, everywhere. There are good people.. there's one lady, she loves me like her son to be honest, she gives me everything […] the things she did for me, nobody would be able to do it. […] But sometimes I – I don’t know why – sometimes I call her and I feel like ‘Okay, this is trust’. And sometimes I feel like don’t trust. […] the lady is nice, I talk to her sometimes, but I don’t trust nobody. No one. It doesn’t matter who they are. […] I am really happy. I love her*.* (Habibullah)

### Theme Two: The Roots of Coping

This theme describes the contexts within which the foundations of coping were learned and the significant figures in participants’ journeys in learning ways of coping. Of significance, discussions of early years were limited by participants’ difficulties in remembering aspects of their childhood and readiness to share difficult experiences. For example, Farhad expressed: “*the most difficult days that I had in my childhood, I can’t share it with you, to be honest*”.

I don’t remember a lot about my childhood, but when I came here I was very wounded. I was very very hurt and like, you know, damaged. […] part of these events made me like forget some of the memories from the past. So I remember the bad things that had happened, but the good ones, whatever, in the past, I can’t remember most of them. (Jamal)

#### Preoccupied Parents

Some participants grew up with parents who shielded them from stress and offered comfort, but who were preoccupied with fear, worry, work or raising a large family:

My father was abducted and went missing, and since then my mother did not allow me to go outside. […] My brother left when I was very young and came here, and I really missed at the time. […] I used to play marbles with him and then there was no one. Sometimes my mum would come and play with me, but not as often as my brother did*.* (Hamid)

Although some described instances of being comforted by parents, only two spoke about being advised by their parents to keep busy and not be alone to manage emotions:

[My parents] did say like, kind of like, “be busy with yourself, don’t leave yourself alone”, like “do something like doing things, like for example cooking, cricket – playing cricket, go to gym, talk to, you know, your friends”. (Zahir)

#### Challenges to Emotional, Physical and Relational Safety

Some participants expressed that they would not share difficulties with their parents or other close relations when they were younger: “*I was hiding a lot of stuff from everyone*” (Yousuf). Many described growing up in a context of limited emotional safety, relating to cultural norms but also parental capacity in the wider context of fear and survival experienced by some parents as described above. Participants spoke of emotional expression not being encouraged, cultural expectations of boys, children's voices not marked as equal, and not bringing shame to the family. For example, Mohammad expressed, “*I would do anything just to impress Dad or Mum*” and Said relayed, “*Don’t think I shared with anyone, including my mother.* […] *She would beat me if I said I had a problem or something*”.

One of the reasons sometimes I suppress my emotions is because it's from a young age, like going to school and stuff, I kept, you know, whatever happened during the day to myself. You know, I don’t share. For example, at school I had a fight, but I don’t share it with my family, you know? […] Because over there, they tend to say you have to stand up on your own feet, right? So if you cry as a boy and stuff, it's not a good thing. […] from a young age, I became this person that… “crying makes you weak”. […] So if I had a bad day as well, I just kept it for myself. (Asadullah)

Some spoke about receiving love and affection from their fathers, but for most this was received from their mothers and fathers were more authoritarian and could be violent. For example, Mohammad shared: “*Mum's approach is very soft*” and “*we could never joke with Dad*”, explaining “*if I had a total breakdown mentally because of my dad,* [my mother] *would hug me, she would do all the love things that makes you feel like you’re loved, you’re important*”.

#### Services as Substitute Parental Figures

Participants reported feelings of warmth and being cared for in their relationships with professionals, describing a reliance and feelings of safety akin to a parent–child relationship. Therapists or caseworkers were often participants’ first port of call for support:

[My caseworker] has done a lot of things for me, he has cared for me and he's very kind. And he's loved me, more than my parents. […] He has done everything and anything for me. […] I’ve never met so kind in my life, and especially a stranger. He's not related to me. He has cared for and done so much for me. (Hamid)

Therapists were described as not only offering psychological support, but also practical support. They were a medium by which participants could build a community, overcome difficulties with cultural orientation and, for some, learn to communicate and make friends. Participants spoke of a journey from dependence on services to independence:

When I came to this country, I was like a blind person. I couldn’t do anything for myself. But he showed me ways of doing things and helped me practically, and I’ve learned quite a lot. And I’m quite independent now […] I’ve shared almost everything, anything I needed help with or just needed to talk about with him. […] I didn’t know how to find a solicitor or how to communicate with the solicitor and he helped me with that. (Ali)

Significantly, therapists were reported to be key figures in helping participants to develop coping strategies:

I’ve been given some techniques by my previous therapist. She said sometimes if you’re really – if you’re feeling really low, you just look around your room and say the names of things loud, look out through the window and that will reassure you that you’re in a safe place. (Hamid)

Input from services was described as transformational by a few participants: “*If I didn’t have those sessions, I’m sure 100% that I would not be coping the way I do now.* […] *I would not be the same person if I didn’t have that help, in every possible way*” (Jamal).

### Theme Three: Coping is Dynamic: The Process of Change

This theme describes how coping is influenced over time by culture and experience, and how new coping strategies can be learned. It speaks to the importance of experiential learning and the effect this has on appraisals and perspectives. This theme also portrays how dual-culture, and religious beliefs and values are internalised and influence coping.

#### The Lens of Experience

Developing strategies to cope was sometimes described as a process of experiential learning, needing to try different things and notice, initiate and experience change: “*if you say anything to me, I’ll not…* *I’ll understand it, acknowledge it, have it in the back of my head. But once I experience myself, then I will 100% put it in my house or my heart*” (Mohammad). For some, developing strategies to cope and seeking help was motivated by “*hitting rock bottom*” (Mohammad).

The first few years, I was like way stressed and then I would always keep in myself, never expressed how I feel. And it really like hit my immune system and […] I thought, no, it's not the good way I think. So, yeah, I started seeing a therapist. (Yousuf)

#### The Importance of Perspective

Participants described shifts in the difficulties they experience and how difficulties are viewed, responding to changes to contextual factors (e.g., immigration stressors diminishing after visas granted). For example, Ali, for whom family unification was possible, shared “*Before my family came over here, I really needed somebody to talk to and share how I felt. But since that they have come here, I can talk to them and it really helps*”. Difficulties were also described as changing naturally as time passes and lessening in response to receiving support:

At the time, like when I was coming to see [my therapist], I was very close to what happened in the past. So it was really difficult. […] whatever I went through, I will talk to her and she will give me advice and I will follow her advice. And it was a long process. It took 3 years to make me learn how to kind of escape from that situation. (Jamal)

#### Socialisation to Talking

Talking was a new coping strategy for many, described to be learned through individual or group therapy, socialising participants to talking about themselves, being heard and discovering the value of expressing themselves. Therapy also offered an opportunity to listen to others:

Going to therapy here and there and talking, this is just made me like kind of slightly open person. Before I was just keeping everything to myself, you know. Not allowing people, probably “this is going to judge me and that's going to judge me”. (Asadullah)

A shift was described from being closed to open, as a result of therapy, as well as redirecting the focus from others to oneself. For some, practising talking and sharing emotions generalised to other relationships. For example, Hamid shared, “*I think I started talking to my brother after the therapy*”.

The therapist has helped me by just listening to me first of all, and talking has helped. I’ve learned from her that you know if you talk it helps and also that I need to think more about myself rather than the outside world. (Said)

Opportunities outside of therapy, and certainly in childhood, were reported to be limited by the cultural norms and parental emotional capacity in the context of survival described in Theme 2. For example, Hamid, whose mother kept him at home to protect him, shared: “*I haven’t been with many people in my life or throughout of my life. That's why I haven’t learned much social skills*”. Some of these opportunities may also have been influenced by cultural norms, as Farhad explained: “*We didn’t even like talk to our elders, nothing, we were just listening, that's all*”.

#### Inheriting and Internalising Religion

Religion was described as social and culturally important, passed on to children by parents and families. The significance of religion, and of religious rituals, was described as changing over time, as it was internalised and owned moving from childhood to adulthood:

I’m from Muslim family, obviously, and religion has been, you know… It's kind of bread and butter of our family, yeah. And society. So yeah, it's been part of my life all the time. […] as you grow, then you value the religion more in your life because initially like 16–17–18, you’re praying but you’re not praying wholeheartedly, you know, you’re just praying for the sake of praying. You know… But yeah, as you grow and then you become more mature, you understand that there is something behind a religion. (Asadullah)

#### Cultural Dislocation, Adaption and Identity Development

Cultural identity appeared to be closely linked to ways of coping and appraisals of types of coping strategies, intersecting with age, religion and gender. The integration of cultural roles, values and beliefs, in a context of cultural dislocation and acculturation, appeared to confuse identity development and coping, especially as these could be incompatible. For example, talking and emotional expression were highlighted to be actively discouraged and socially unaccepted in Afghan culture, even within family homes and particularly for boys, which is at odds with the value of talking and therapy highlighted by participants:

In Afghanistan […] mental health there and this kind of thing, no one took it serious. Everyone would probably call you like, you know, crazy talking about it. […] I think culturally, like emotion is not, yeah, taken very serious. […] So to talk about it and think… it's just a waste of time, “what are you talking about?”, you know? Like if you’re having a bad day, difficult day, “come on man”, “what are you complaining, someone who has badder day than you”, so yeah… So it's just like comparing, and all of that… (Asadullah)

## Discussion

This qualitative study aimed to explore the difficulties and coping responses of former URMs from Afghanistan and gather their perceptions of how and from where coping strategies were developed. Reflexive Thematic Analysis of 12 interviews generated three overarching themes: “*Difficulties and coping: a series of cumulative challenges and vicious cycles*”; “*Roots to coping*”; and “*Coping is dynamic: the process of change*”. Participant narratives highlight ongoing vulnerability and mental health difficulties, consistent with quantitative evidence that URMs can suffer from mental health difficulties for years after resettlement (Vervliet et al., 2014). The findings also indicate the influence early years and relationships can have on the ways coping strategies may be appraised and developed and the mechanisms by which experiences of early attachment, dual cultural norms and identity could influence coping.

Participants reported multiple and compounding difficulties, chiming with research finding high prevalence of emotional and behavioural problems among Afghan URMs (Bronstein et al., 2012; Bronstein et al., 2013). Difficulties were often present in a triad of physical/psychosomatic pain, mental health difficulties and relational challenges. This is consistent with qualitative research among Afghan and Kurdish refugees in Australia, which puts forward that rumination in the context of loneliness can rekindle traumatic memories and prolong mental health difficulties long after resettlement (Sulaiman-Hill & Thompson, 2012). A reliance on avoidance and distraction was reported by participants as a coping strategy for difficulties relating to haunting thoughts, echoing previous findings (Behrendt et al., 2023; Ní Raghallaigh & Gilligan, 2010) and aligning with participant reports of cultural norms and expectations regarding emotional expression and masculine ideals. Religion was also a significant anchor for coping for some. These findings offer support for culturally specific influences and contexts in which coping strategies are developed and how difficulties and coping are expressed (Eggerman & Panter-Brick, 2010; Ungar, 2006; Ungar et al., 2007).

The present study sheds light on resilience mechanisms and barriers to resources and approaches being used and being effective, revealing how coping strategies may be limited by difficulties and even exacerbate further challenges. Although all participants have experienced violence and threat to life or livelihood, in addition to parental loss or separation, it was not in the scope of the present study to enquire about these experiences or their consequences. Nonetheless, the confusion and fragmentation observed in the interviews resemble symptoms of PTSD and complex PTSD, for example loss of memory, lack of internal integration or disruption to self-organisation, and emotional lability, in addition to lack of experienced safety, problems forming relationships, and negative self-concept (Brewin et al., 2017). At times, these could be observed to affect participants’ sense of wellbeing and interact with coping strategies, impeding their use, or limiting their effectiveness. In this vein, some participants were equipped with helpful coping strategies and resources and yet continued to experience difficulties and distress, echoing previous findings (Sutton et al., 2006), and perhaps reflecting the severity of their trauma and difficulties exacerbated by current difficult life circumstances. These findings might help explain why the relationships between coping strategies and mental health outcomes have been found to be nuanced in conflict-affected adults (Seguin & Roberts, 2017).

Ongoing mental health difficulties have been associated with challenges with social integration and belonging among refugees with PTSD in previous research (Schick et al., 2016). A need for, and preoccupation with, social connection was apparent in participant narratives, at times as means for distraction, to tackle loneliness and to experience belonging. While some participants had established trusted relationships outside of professional services, others found this challenging or undesirable due to mistrust and uncertainty in connecting with others. The boundaries and assigned roles within relationships with professionals and the prioritisation of safety and trust may help explain the contrasting experiences described of building relationships with friends compared to therapists. Interestingly, few participants mentioned relationships with siblings, other than to express worry or loss. Siblings, according to Juliet Mitchell′s psychoanalytic concept of “the Law of the Mother”, are mediums by which social worlds are learnt and navigated, extending over time to other lateral relationships such as cousins or friends (Mitchell, 2022). In addition to the relational insecurity commonly experienced following trauma (Brewin et al., 2017), the absence of reports of emotional closeness with siblings and friends growing up due to loss, separation/rejection or prioritisation of survival may help explain the thwarted social connection and challenges with social skills experienced by some participants.

Negotiating identities following displacement has been intrinsically linked with psychosocial wellbeing and increased risk of suicide among migrant and refugee youth following resettlement (Mude & Mwanri, 2020). Participants were recruited at a time of transition, represented by early adulthood and dual cultural integration, in which they were learning, and unlearning, ways to cope. At times, participants’ identities and coping strategies were observed to be confused and fragmented, possibly reflecting complex intersectionality shaped by cultural norms and expectations of age, gender, religion and emotional expression in both Afghan and British cultures. While upholding cultural values has been posited to play a vital role in shaping social identity, cohesion, and a sense of hope, failing to meet cultural expectations can lead to significant psychological and social distress (Eggerman & Panter-Brick, 2010). The additional complexity of dual culture integration may obstruct the ability to learn or use coping strategies and validate emotional experiences (i.e., cognitive incongruence), and contribute to feelings of isolation, shame and helplessness. Prolonged avoidance of experiencing emotions and lack of practice and discomfort relating to emotional expression in the context of cultural norms and beliefs in Afghanistan, in particular, sat at odds with the importance of talking and connection described by participants. This is documented in previous research as a barrier to accessing mental health support for URMs, and has implications for how therapeutic interventions may be presented and approached (Demazure et al., 2022; Motti-Stefanidi, 2018). Further exploration of identity formation and conflict may be valuable in light of these findings.

Understanding how attachment systems contribute to resilience processes among refugee youth has been highlighted as an especially useful area of research, particularly given the well documented risks of peri-migration stressors and traumatic experiences (Juang et al., 2018). Cultural norms and familial relational dynamics, including critical parenting exacerbated by fear and political instability, may hinder emotional expression and challenge secure attachment, as suggested by participants keeping difficulties to themselves and feeling unsafe and uncertain to express emotions with primary caregivers. Decades of conflict and instability in Afghanistan have profoundly affected all areas of society, including religious practices, education and financial security, and laid the foundation for widespread mistrust and intergenerational trauma (Panter-Brick, 2023). Parental mental health difficulties and trauma have been shown to impact parenting styles, parent–child relationships and child mental health outcomes (Betancourt et al., 2015; Daud et al., 2005; Eruyar et al., 2018; Panter-Brick et al., 2014; Scharpf et al., 2023). Participant narratives suggest there may be further influences of cultural beliefs and values, for example high expectations of children or mental health stigma, as well as fear, hypervigilance, and limited parental emotional availability. Participants rarely spoke of parents as significant figures in supporting them to cope with difficulties, emphasising instead the central role for professional services, stepping in as parental figures, in supporting them to navigate independence and develop coping strategies by providing a secure base.

## Strengths and Limitations

The present study provides qualitative exploration of difficulties and coping among Afghan former URMs, an under-researched group (Alemi et al., 2014), providing a valuable contribution to the evidence base. This is the first study among refugee samples seeking to gain perspectives on how and from where coping strategies are developed. It captures the experiences and perspectives of participants from one cultural background, reducing heterogeneity, and adheres to a quality checklist (Tong et al., 2007), ensuring important aspects of the research are reported. Given the known considerations and challenges with recruitment of refugee populations (Miller, 2004), the present study recruited a modest sample suitable for RTA (Terry et al., 2017). The study invested considerable efforts to include Afghan community members in various phases of the research process, highlighted to be vital in research involving Afghan refugees (Alemi et al., 2014), which informed the design and supported with data interpretation.

Nonetheless, the purposive sampling strategy used can increase the likelihood of selection bias. For example, all participants accessed psychotherapy and were recruited via the same service, which may have prompted participants to speak about professional support to a greater extent than if other recruitment strategies had been used. Although early attachment experiences are discussed in the present study, attachment was not explicitly measured, limiting understanding of the patterns and mechanisms at play linking attachment to coping. In addition, only male participants took part, despite efforts to recruit a mixed-sex sample. Future research should also aim to recruit females, whose needs may be different to males (Fazel et al., 2015) and whose attachment experiences and cultural roles may differentially influence coping. Finally, this study did not capture information or perspectives regarding age of separation or displacement, which may help to better understand how early experiences, developmental difficulties and attachment contribute to coping.

## Implications and Conclusions

The present findings underscore the importance of services offering scalable, long-term, and tailored interventions for refugees (Henkelmann et al., 2020). Given the multitude of challenges described by participants, holistic, person-centred care is warranted, considering the interplay of mental and physical health and identity formation, and subsequent effects on coping. Sensitivity to cultural influences is required by services to promote coping and consider barriers to these being adopted or employed (Scharpf et al., 2021). Trauma- and attachment-informed care is essential, given the relational insecurity and broad impact of trauma portrayed by participants. Interventions focusing on emotion regulation and promoting coping, such as “Problem Management” (Perera & Lavdas, 2020) or “Skills-Training of Affect Regulation – A Culture-sensitive Approach” (Koch et al., 2020), which have been successfully trialled among Afghan refugee groups, may be especially useful to draw from.

Our findings, like others before us, emphasise coping strategies centred on avoidance and distraction (Behrendt et al., 2023; Ní Raghallaigh & Gilligan, 2010), whilst also highlighting the value of social connection, belonging and a safe base. The juxtaposition of loneliness and connection, accompanied by uncertainty and mistrust, underscores the need to prioritise fostering a sense of belonging through community-focused interventions and activities. Customised attachment-based interventions, tailored to Afghan populations and culture (e.g., El-Khani et al., 2021), may benefit staff working with URMs, whose attachment relationships may be complex as a result of childhood adversity and parental separation or rejection. Professional support, described by participants to foster feelings of relational safety, could be conceived as an antidote to neglect and to the development of emotionally avoidant defences. Nonetheless, the significance of professional support for coping and well-being, as described by participants, raises concerns for the many URMs and refugees who do not have access to trauma interventions or mental health support services (Sanchez-Cao et al., 2013; Satinsky et al., 2019).
